# Burn inhalation injury and intubation with dexamethasone-eluting endotracheal tubes modulate local microbiome and alter airway inflammation

**DOI:** 10.3389/fbioe.2025.1524013

**Published:** 2025-02-26

**Authors:** Gabriela Gonzales, Ronit Malka, Rena Bizios, Gregory R. Dion, Teja Guda

**Affiliations:** ^1^ Department of Biomedical Engineering and Chemical Engineering, University of Texas at San Antonio, San Antonio, TX, United States; ^2^ Department of Otolaryngology – Head and Neck Surgery, Brooke Army Medical Center JBSA Fort Sam Houston, San Antonio, TX, United States; ^3^ Department of Otolaryngology – Head and Neck Surgery, University of Cincinnati, Cincinnati, OH, United States; ^4^ Department of Cell Systems and Anatomy, University of Texas Health San Antonio, San Antonio, TX, United States

**Keywords:** inhalation injury, upper airway, microbiome, inflammation, endotracheal tube

## Abstract

**Background:**

Inhalation injuries, caused by exposure to extreme heat and chemical irritants, lead to complications with speaking, swallowing, and breathing. This study investigates the effects of thermal injury and endotracheal tube (ETT) placement on the airway microbiome and inflammatory response. A secondary aim is to assess the impact of localized dexamethasone delivery via a drug-eluting ETT to reduce laryngeal scarring.

**Methods:**

Inhalation injury was developed in swine by administering heated air (150°C–160°C) under endoscopic visualization. Following injury, segments of regular or dexamethasone-loaded endotracheal tubes (ETTs) were placed in the injured airways for 3 or 7 days. Computed tomography (CT) scans were used to assess airway narrowing post-injury. Biofilm formation on the ETTs was investigated using micro-CT and microscopy. The airway microbiome was analyzed via 16S rRNA sequencing. Inflammatory markers were quantified using an immunoassay and macrophage populations in laryngeal tissue were assessed with CD86 and CD206 staining. Tracheal tissues were also histologically examined for epithelial thickness, collagen area, and mucin production.

**Results:**

CT scans confirmed airway narrowing post-injury, particularly around ETT sites. Biofilm formation was more extensive on dexamethasone-coated ETTs at later timepoints. Beta diversity analysis revealed significant shifts in microbial composition related to ETT type (R^2^ = 0.04, p < 0.05) and duration of placement (R^2^ = 0.22, p < 0.05). Differential abundance analysis demonstrated significant positive log fold changes in genera such as *Bergeriella*, *Peptostreptococcus,* and *Bacteriodes* with thermal injury over time. Inflammatory markers IFN-γ, IL-4, and IL-1β were elevated in dexamethasone-ETT groups at 3 days, then decreased by 7 days. Macrophage markers CD86 and CD206 were significantly greater in dexamethasone groups compared to regular ETT groups at 7 days (p = 0.002 and p = 0.0213, respectively). Epithelial thickness was significantly greater with regular ETT placement compared to dexamethasone ETT placement in the burn-injured airway at 3 days (p = 0.027).

**Conclusion:**

Thermal inhalation injury and ETT placement significantly impact airway inflammation, structural integrity, and microbiome composition. Dexamethasone-eluting ETTs, intended to reduce inflammation, increased biofilm formation and elevated cytokine levels, suggesting complex interactions between the drug coating and the host immune response. The airway microbiome shifted significantly with specific taxa thriving in the inflamed environment.

## Introduction

Severe burn injuries are often accompanied by inhalation injuries, involving acute damage to the respiratory system caused by exposure to chemicals and/or heat (Monteiro et al., 2017; Charles et al., 2022). This occurs because of the glottic closure reflex-the instinctive adduction of the vocal folds protecting the lower airways causing the vocal folds themselves to brace the impact of heat exposure. Additionally, insult below the glottis can result from the irritants present in smoke. Such injuries can lead to delayed airway edema often necessitating ventilatory support and generally requiring prophylactic intubation (Foncerrada et al., 2018; Cochran, 2009; Orozco-Peláez, 2018). Acute tissue injury and subsequent use of indwelling medical devices, such as endotracheal tubes (ETTs), disrupts the respiratory mucosal barrier, increasing the risk of infections like pneumonia (Ronkar et al., 2023; Edelman et al., 2007). More severe complications that can arise include acute respiratory distress syndrome (ARDS), or stenosis due to the accumulation of laryngeal or tracheal scar tissue (Bittner and Sheridan, 2023; Jones et al., 2017; Gaissert et al., 1993).

The microbiome of the respiratory tract plays an integral role in maintaining immune homeostasis and influencing disease pathology. Trauma, such as inhalation injury, can lead to dysbiosis between microbes which often results in favor of opportunistic pathogenic microbes (Natalini et al., 2023). Bacterial infections, often the precursor to pneumonia, are frequently identified through histological examination and culturing respiratory mucous secretions (Torres et al., 2016). However, these methods can miss rare or less abundant taxa that can play significant roles in the development of chronic diseases. To address these limitations, 16S rRNA sequencing has emerged as a valuable technique for providing a comprehensive view of the bacterial species present. This method has been extensively used to study bacteria associated with inflammatory airway diseases such as chronic obstructive pulmonary disease (COPD) and asthma, revealing taxa that thrive in inflamed microenvironments (Ramsheh et al., 2021; Wang et al., 2016; Leiten et al., 2020; Millares et al., 2015; Pragman et al., 2012; Tangedal et al., 2019; Marri et al., 2013; Morimoto et al., 2024). Despite these advances, our current knowledge of the airway microbiome’s precise role in inhalation injury sequelae remains limited, indicating a significant gap that requires further investigation (Dyamenahalli et al., 2019).

In parallel, airway inflammation is a natural response to injury and a part of the healing process. Following damage, immune cells are recruited to the site of injury to remove pathogens and initiate tissue repair (Aghasafari et al., 2019; Huber-Lang et al., 2018). This acute inflammatory response is characterized by the release of various cytokines and chemokines that help to regulate the body’s defenses but, if persistent, can exacerbate the injury and delay healing. Chronic inflammation is often associated with an imbalance between pro-inflammatory and anti-inflammatory mediators such as different macrophage phenotypes (Chang-Hoon and Eun Young, 2018; Koh and DiPietro, 2011). Understanding the mechanisms underlying immune response in inhalational injury can contribute towards the development of effective treatments for these conditions, potentially preventing long-term complications.

Airway mucins are major components of mucus, a gel-like substance that lines the mucosal epithelium and protects the airway from inhaled pathogens and toxins (Song et al., 2020). Mucus facilitates the expulsion of irritants through mucociliary clearance or coughing. In healthy individuals, mucus production is continuous, maintaining a balanced and protective layer (Pangeni et al., 2023). Biofilms, however, are structured communities of microorganisms encased in a self-produced extracellular matrix (Hall-Stoodley and McCoy, 2022; Domingue et al., 2020). They often adhere to surfaces such as endotracheal tubes and can stimulate the production of additional mucins, potentially leading to hypersecretion (Mishra et al., 2024; Powell et al., 2018). Both excessive mucus and biofilm presence can exacerbate inflammatory responses, as they create a favorable environment for sustained immune activation and persistent microbial colonization (Moser et al., 2017; Fahy and Dickey, 2010). Inhalation injuries can cause pathophysiologic changes, including thickened mucus, loss of surfactant, and impaired mucociliary function (Foncerrada et al., 2018; Cox et al., 2008). Exploring the alterations in mucus secretion and ETT biofilm formation associated with thermal inhalation injury could thus provide valuable insights into the inflammatory response to design timely interventions and corticosteroid or other interventional therapy leading to potential improvements in patient outcomes and quality of care.

The objective of this study was to investigate the effects of thermal inhalation injury and ETT placement on the interdependency between the microbiome and inflammatory response in the upper airway. A secondary objective was to evaluate the impact of localized delivery of dexamethasone on these outcomes using a drug-eluting ETT to prevent excessive laryngeal scarring and stenosis.

## Materials and methods

### Study design

**FIGURE 1:**
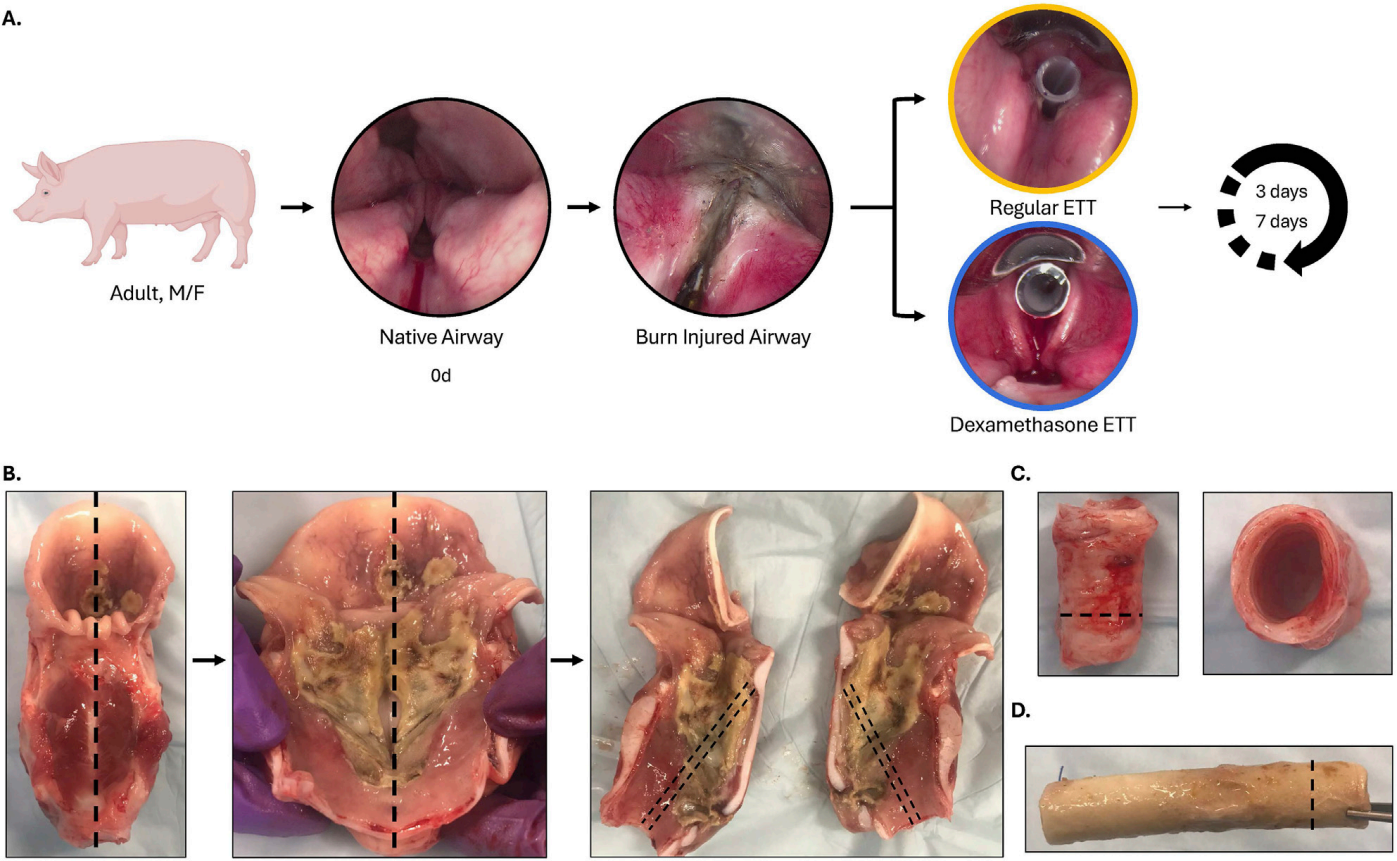
Experimental overview **(A)** Inhalation injury was simulated under endoscopic visualization, and a 5 cm segment of regular (uncoated ETTs) or dexamethasone-loaded PCL fiber-coated ETTs were placed for 3 or 7 days. **(B)** The larynx was extracted immediately after euthanasia and sectioned in the sagittal plane, with care taken to preserve the anterior commissure. Sections were taken along the mid-region of the vocal fold for histological evaluation. **(C)** Sections of the trachea (side view/top view) with an inset line illustrating sections selected for histological evaluation and **(D)** endotracheal tube after the end of study demonstrating biofilm formation, with an inset line representing the sections taken for histological evaluation.

The current study was approved by the U.S. Air Force 59th Medical Wing Institutional Animal Care and Use Committee (protocol FWH20210102AR). An experimental overview is presented in Figure 1. Laryngeal thermal injury was simulated in Yorkshire crossbred swine under endoscopic visualization. Endotracheal tube (ETT) segments, with or without a dexamethasone-eluting electrospun fiber coating, were then placed for either 3 days (n = 5) or 7 days (n = 4). Biofilm formation and bacterial adhesion were examined using scanning electron microscopy (SEM), micro-computed tomography (µCT), and histology. Changes in the microbiome following injury and ETT placement were assessed through 16S rRNA sequencing. The inflammatory response of the upper airway was evaluated using immunohistochemistry and immunoassays. Additionally, histological evaluation of the trachea was conducted to assess epithelial changes.

### Endotracheal tube coating

ETTs were coated via electrospinning as previously described by our group (Gonzales et al., 2024). Briefly, polycaprolactone (PCL) (Mw = 80,000) was dissolved in chloroform (15:85 w/w) and dexamethasone sodium phosphate was added to the homogeneous mixture at a concentration of 10% (PCL:Drug) along with its solvent ethanol.

The solution was then loaded into a Luer Lock syringe and dispensed through an 18G blunt tip needle using a syringe pump (Pump11 Elite, Harvard Apparatus, Holliston, MA) at a rate of 1.8 mL/hr. A 5 cm section of section of an ETT (7–0, Aircare^®^) was positioned on a rotating rod (300 rpm) 20 cm below the needle tip where a voltage of 20 kV was applied (Gamma High Voltage Research, Ormond Beach, FL). The coated ETTs were subsequently sterilized with ethylene oxide before use. Using only a section of the ETT was intended to enable the animals to maintain normal activity throughout the study instead of being sedated and intubated for extended periods. Unless otherwise specified, chemicals were sourced from Sigma-Aldrich (St. Louis, MO).

### Laryngeal inhalation injury simulation and ETT placement

Animals were anesthetized with intramuscular Telazol^®^ and Ketamine (2.2 mg/kg) and maintained on 0.5%–5% isoflurane during the procedure. Pain relief was provided with intramuscular Buprenorphine (0.01–0.05 mg/kg). Laryngeal burn injuries were created as previously described (Malka et al., 2023) by administering heated air (150°C–160°C) to the larynx of spontaneously breathing animals in 30-s intervals for 5 min at a rate of 5–10 mL/min under endoscopic visualization.

During direct laryngoscopy, a second surgeon assisted with ETT segment placement. The neck area was prepped with povidone-iodine solution, and 1% lidocaine with epinephrine was injected at the level of the cricoid cartilage. A 3–4 cm midline neck incision was made, and dissection was performed to expose the first three to four tracheal rings. Two 16-gauge angiocatheter needles were inserted into the larynx, one through the cricothyroid membrane and the other between the first and second tracheal rings. A urologic snare was passed through each angiocatheter and out through the mouth. The surgeon passed a 2–0 polypropylene suture through the distal end of the ETT segment, securing each end of the suture to the snares, which were then retracted back through the angiocatheters and the neck. The sutures were pulled taut while the ETT segment was positioned in the larynx, with the lower edge in the subglottis and the upper edge just past the epiglottis. Postoperatively, each animal was monitored hourly for the first 4 h, every 4 h for the next 20 h, and at least twice daily until the end of the study to ensure proper respiratory status.

Euthanasia was carried out with intravenous pentobarbital (100 mg/kg) and confirmed by monitoring vital signs according to institutional protocols. Animals experiencing distress or illness were euthanized earlier than the scheduled postoperative date. The larynx was extracted immediately post-euthanasia, sectioned in the sagittal plane, and frozen at −80°C for subsequent analysis.

### Large animal computed tomography

Immediately after the placement of ETTs, animals underwent a computed tomography (CT) scan (Toshiba America Medical Systems Inc., Tustin, CA) with 0.625 mm slice thickness and images were saved in digital imaging and communications in medicine (DICOM) format (Carlisle et al., 2019). Raw images were input into Mimics (Materialise NV, Leuven, Belguim) and the airway region where the ETT was placed was reconstructed into a 3D model. Briefly, a new mask was created based on a “custom” range of Hounsfield Unit (HU) values from −650 to −200 to highlight the airway tissue. From this airway mask, a 3D part was reconstructed and the surface area (SA, mm^2^) and volume (V, mm^3^) of the part were obtained. The SA/V ratio was then measured as an imaging biomarker for luminal narrowing.

### Biofilm quantification

Following euthanasia, the extracted ETTs were placed in 10% formalin. The samples were stained with phosphotungstic acid (PTA) and imaged with micro computed tomography (µCT) (Skyscan 1,076, Bruker, Billerica, MA). The ETT scans were imported into Mimics (Materialise NV, Leuven, Belguim) where the tube, biofilm, and guide wire were spatially distinguished. The volume, area, and surface area of the biofilm inclusions were determined from the segmented 3D models. Following µCT scanning, a section (∼1 cm) of the ETT was set aside for histological analysis and SEM.

### Gram staining of ETT

ETT sections were embedded in optimal cutting temperature compound (Scigen Tissue Plus O.C.T. Compound, Thermo Fisher Scientific, Waltham, MA) and stored at −80°C. Samples were sectioned with a cryostat (Epredia™ NX70, Kalamazoo, MI) to a thickness of 20 μm, thaw-mounted onto glass slides, and Gram stained (Harleco Gram Stain Set, Sigma-Aldrich, St. Louis, MO). The prepared slide was flooded with crystal violet solution (1 min) and gently rinsed with distilled water. The slides were then flooded with gram iodine solution (1 min) and rinsed gently with distilled water. The samples were decolorized until the solution ran colorless from the slide and washed with distilled water. Finally, the slide was flooded with safranin stain (1 min), rinsed gently with distilled water, and mounted with permount mounting medium (ThermoFisher Scientific, Waltham, MA).

### ETT surface characterization

SEM was used to evaluate the morphology of the ETT surfaces after placement. All specimens were dried using a critical point drier (Leica, Wetzlar, Germany), sputter coated with silver-palladium (Cressington Scientific Instruments, Watford, United Kingdom), and imaged under 2 kV applied voltage at 1,000x magnification using a Zeiss Crossbeam 340 Focused Ion Beam (FIB)-SEM (ZEISS, Oberkochen, Germany).

### 16S rRNA sequencing

At each laryngoscopy (initial and end of study), a swab of the larynx/trachea was collected and stored at −80°C. In addition, the surface of the ETT was swabbed following their removal. The samples were given to the UTSA Genomics Core Facility to be amplified and sequenced. In short, a ZymoBIOMICS™ DNA Miniprep kit (Zymo Research) was used to extract bacterial DNA from the samples according to the manufacturer’s instructions. Following bacterial DNA isolation, the V3-V4 region of the 16S rRNA gene was amplified using universal primer sets 341F and 805R. Sequences were obtained on an Illumina MiSeq platform (Illumina, San Diego, CA) in a 2 × 300 bp paired-end run using a MiSeq v3 kit and following the 16S Metagenomic Sequencing Library Preparation protocol.

The raw sequencing reads were processed using R Studio (v2021.9.1.372, http://www.rstudio.com/). Cutadapt (v4.1) was used for removal of primers from the reads and the DADA2 pipeline (v1.16) was used for subsequent processing (Callahan et al., 2016; Martin, 2011). Briefly, the demultiplexed fastq files for each sample were filtered and trimmed to remove low-quality sequences and run through DADA2’s core denoising algorithm to determine inferred composition of the samples. The forward and reverse reads were merged, an amplicon sequence variant (ASV) table was constructed, and chimeras were removed. Species-level taxonomy was assigned to the sequence variants using the Silva (v138.1) database (Quast et al., 2013). The R packages phyloseq, vegan, and ggplot2 were used for downstream analysis and visualization of the sequencing data (McMurdie and Holmes, 2013).

### Immunohistochemistry

Laryngeal tissues were sectioned into a 5 mm thick section along the mid-region of the vocal fold, fixed in 4% formalin overnight, and mounted in disposable embedding molds with O.C.T. Tissue samples were stored at −80°C prior to sectioning with a cryostat to a thickness of 14 µm and thaw-mounting onto glass slides. Slides were thawed at room temperature for 10 min and washed in sterile PBS to rehydrate (2 times, 10 min). They were then permeabilized in 1% goat serum and 0.4% TritonX100 in PBS (2 times, 10 min). Sections were blocked with 5% goat serum in PBS for 1 h at room temperature and placed to air dry for 5 min. Tissue sections were incubated in antibodies anti-CD86 (1:100, ab269587, Abcam, Cambridge, MA) and anti-CD206 (1:50, ab8918, Abcam, Cambridge, MA) in 5% goat serum for 2 h at room temperature. Following incubation, sections were washed with PBS (3 times, 5 min) and incubated in a secondary antibodies Alexa 647 (1:1,000, Cat. A21244, ThermoFisher Scientific, Waltham, MA) and Alexa 546 (1:1,000, Cat. A110033, ThermoFisher Scientific, Waltham, MA) for 1 h at room temperature. Slides were washed in PBS (3 times, 5 min), counterstained with DAPI (Cat. R37606, Invitrogen, Waltham, MA), washed again, and mounted with Prolong Diamond Antifade Mountant (Cat. P36970, Invitrogen, Waltham, MA). The stained slides were imaged using an Operetta CLS (PerkinElmer, Ausin, TX) with a water immersion objective lens, in non-confocal mode at ×20 magnification. Images were analyzed using Harmony 4.9 PhenoLOGIC software (PerkinElmer). First, the nuclei were identified to determine the approximate number of cells. Then, the intensity properties for Alexa 647 and Alexa 546 were used to quantify the number of CD86^+^ and CD206+ cells and reported as a percentage of number of positive cells per total number of cells. 4 regions from the epithelium and 4 regions from the vocalis muscle were analyzed for each sample.

### Cytokine and chemokine immunoassay

The presence of IFN-α, IFN-γ, IL-1β, IL-10, IL-12/IL-23p40, IL-4, IL-6, IL-8 (CXCL8), and TNF-α were determined using a ProcartaPlex™ Porcine Panel (Cat. EPX090-60829-901, Invitrogen, Waltham, MA) according to the manufacturer’s protocol. Biopsy punches of the trachea were taken and 500 µL of cell lysis buffer (CellLytic™ MT, Sigma Aldrich, St. Louis, MO) was added per 100 mg of tissue along with 10 µL protease inhibitor (ThermoFisher Scientific, Waltham, MA) per 1 mL of lysis reagent. The tissue was homogenized and centrifuged at 14,000 × g for 15 min at 4°C. For tissue homogenates, 25 µL of Universal Assay Buffer was added to 25 µL of the sample to each well. The concentration was measured by running the samples on a Bio-Plex^®^ 200 system using Bio-Plex manager software (Bio-Rad, Hercules, CA).

### Histological analysis

Frozen tracheas and mounted in disposable embedding molds with O.C.T. Tissue samples were sectioned with a cryostat to a thickness of 14 µm and thaw-mounted onto glass slides. Slides were stained with hematoxylin and eosin (H&E) (Richard-Allan Scientific™ Signature Series™ Stains, Thermo Fisher Scientific, Waltham, MA), Masson’s trichrome (Newcomer Supply, Middleton, WI), and Alican Blue/Periodic Acid Schiff (Newcomer Supply, Middleton, WI) according to the manufacturer’s protocol. A Motic EasyScan Pro 6 Slide Scanner (Motic Instruments, Schertz, TX) was used to image slides at 40X.

Epithelial thickness along the trachea was measured from Masson’s trichrome stained samples using ImageJ (Version 1.53k, National Institute of Health, United States. The area of collagen, expressed as percentage of the total area, also based on Masson’s trichrome was determined in ImageJ. Briefly, images were deconvoluted using the color deconvolution2 plugin and the percentage of pixels above the threshold (0–105, min-max) in the area above the tracheal cartilage was evaluated using the blue component representative of collagen (Landini et al., 2021). The color deconvolution2 plugin was also used to determine percentage of positive staining for Alcian Blue and Periodic Acid Schiff. Results were reported as a ratio of Alcian Blue to Periodic Acid Schiff (AB: PAS).

### Statistical analysis

Comparison of SA/V ratio between non-injured and burn injured airways with ETT placement was analyzed with an unpaired t-test. For comparing ETT biofilm volume, alpha diversity, macrophage markers, cytokine levels and histological outcomes across ETT type and duration of placement, statistical analysis was performed using a two-way ANOVA followed by Tukey’s host hoc test. Alpha diversity was evaluated with Shannon and Chao1 indices to estimate within-sample evenness and richness. The beta diversity describing diversity across samples was assessed with principal coordinate analysis (PCoA) based on the Bray-Curtis dissimilarity index. Statistical differences among groups were determined by permutational analysis of variance (PERMANOVA) using the function adonis from the vegan R package. Differential abundance analysis was performed with ANCOM-BC2 (Lin and Peddada, 2020; Lin et al., 2022) between the regular ETT groups versus dexamethasone ETT groups and 0 days swabs versus swabs taken at 3 and 7 days following the end of study. Significant differences were determined at p < 0.05 for all statistical measures.

## Results

### Surface area-to-volume ratio

**FIGURE 2:**
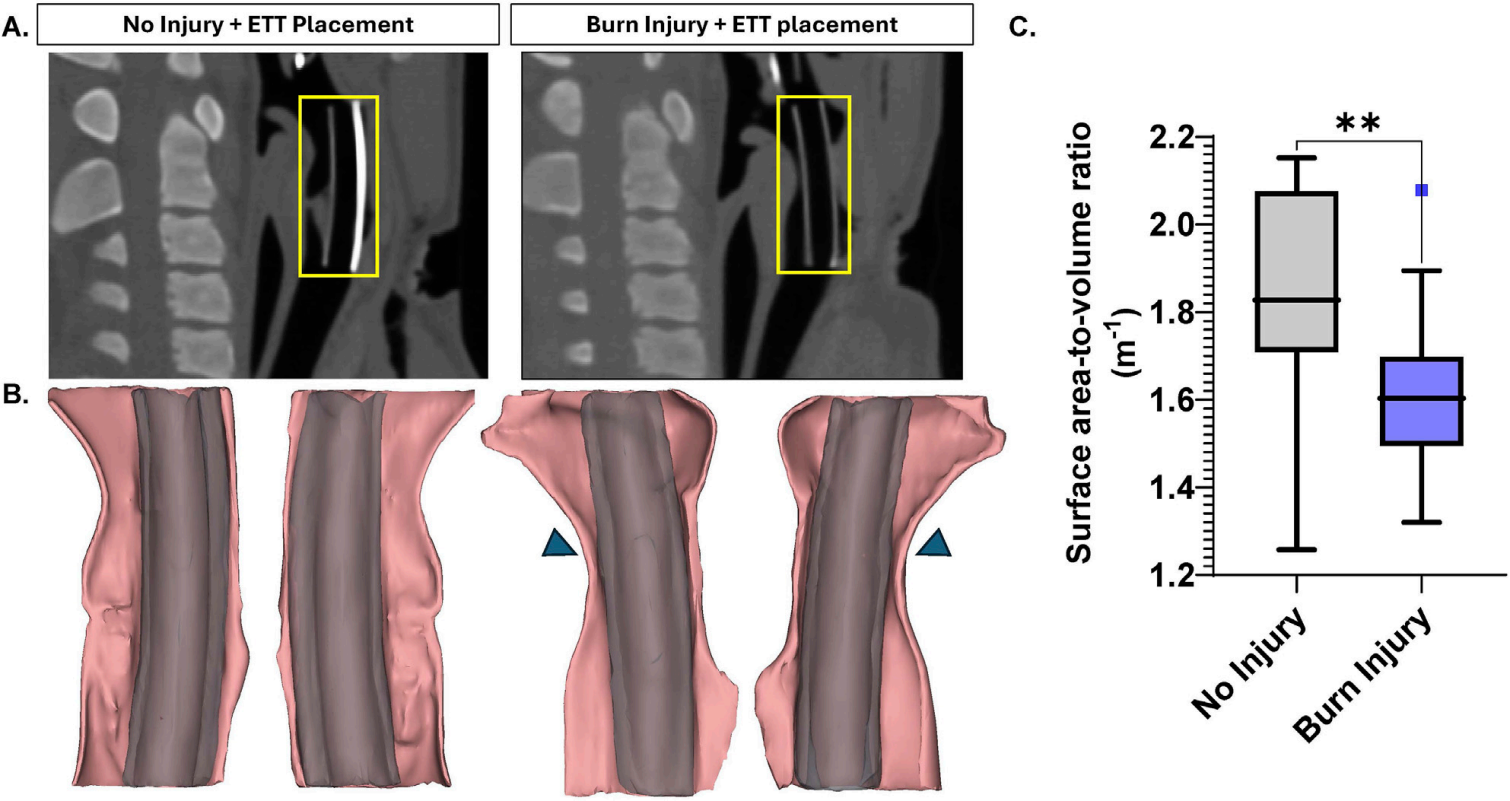
Large Animal CT Analysis **(A)** Sagittal view of non-injured and burn injured airways after ETT placement with an inset (yellow) illustrating region of interest where the 5 cm segment was placed, selected for 3D modeling **(B)** 3D reconstructions with inset arrows indicating areas of airway narrowing **(C)** Surface area-to-volume ratio measurements for non-injured and burn injured airways. Statistically significant differences are indicated by *<0.05, **<0.01, ***<0.001, and ****<0.0001.

Figure 2A presents a sagittal view of the airway following ETT placement in both uninjured and burn injured airways. The 3D renderings of the regions where the ETT sections were placed demonstrated airway narrowing due to burn injury (Figure 2B). Statistically, these differences were significant, with the SA/V ratio being significantly greater in the uninjured airway (1.84 ± 0.06 m^−1^) compared to the burn injured airway (1.61 ± 0.05 m^−1^, p = 0.003) (Figure 2C).

### Endotracheal tube biofilm inclusions

**FIGURE 3:**
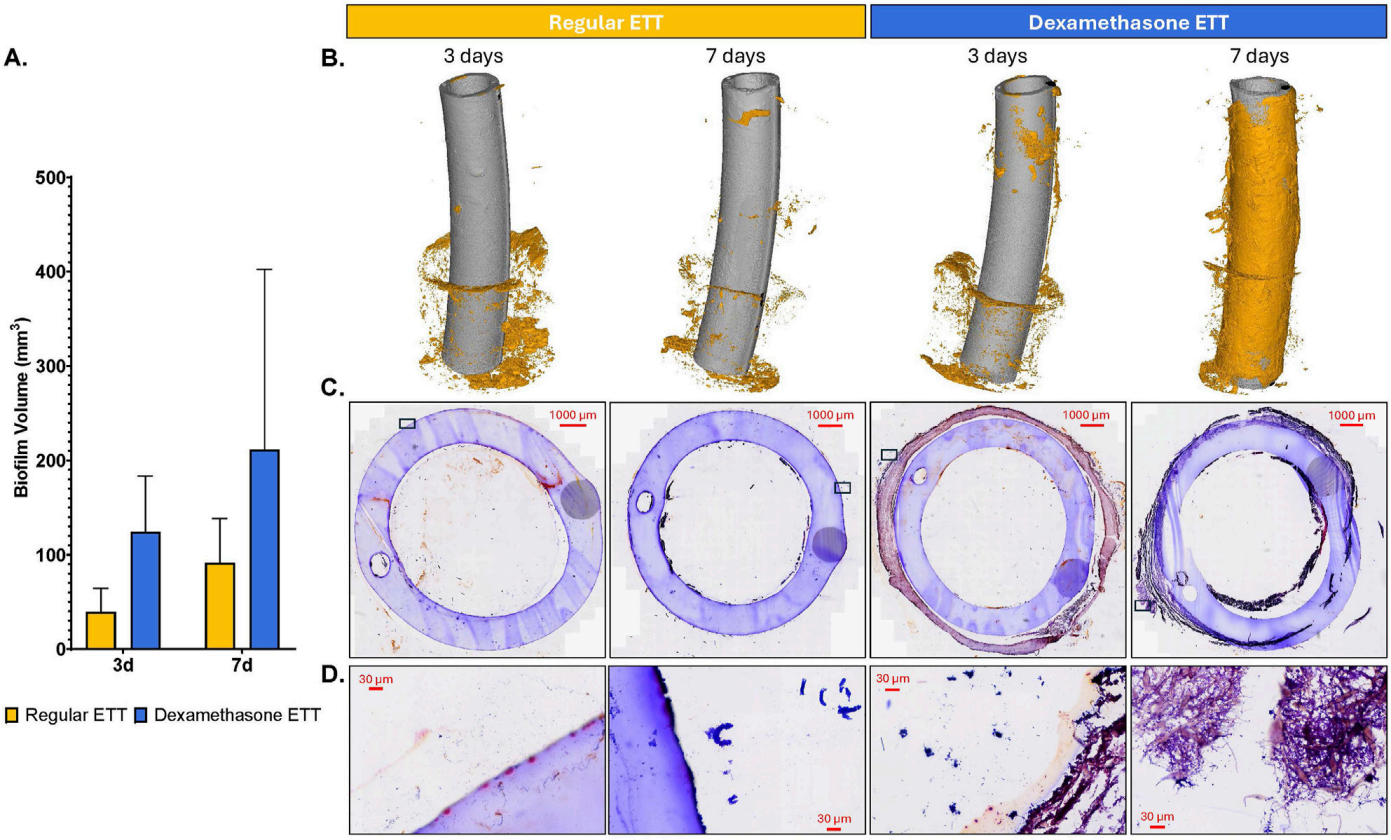
Endotracheal tube Analysis **(A)** Quantification of biofilm volume (mm^3^). **(B)** 3D composites constructed from µCT scans of regular and dexamethasone-coated ETTs, demonstrating biofilm inclusions at the end of study. **(C, D)** Gram-stained sections of endotracheal tubes for regular and dexamethasone coated ETTs at 3 and 7 days.

**FIGURE 4:**
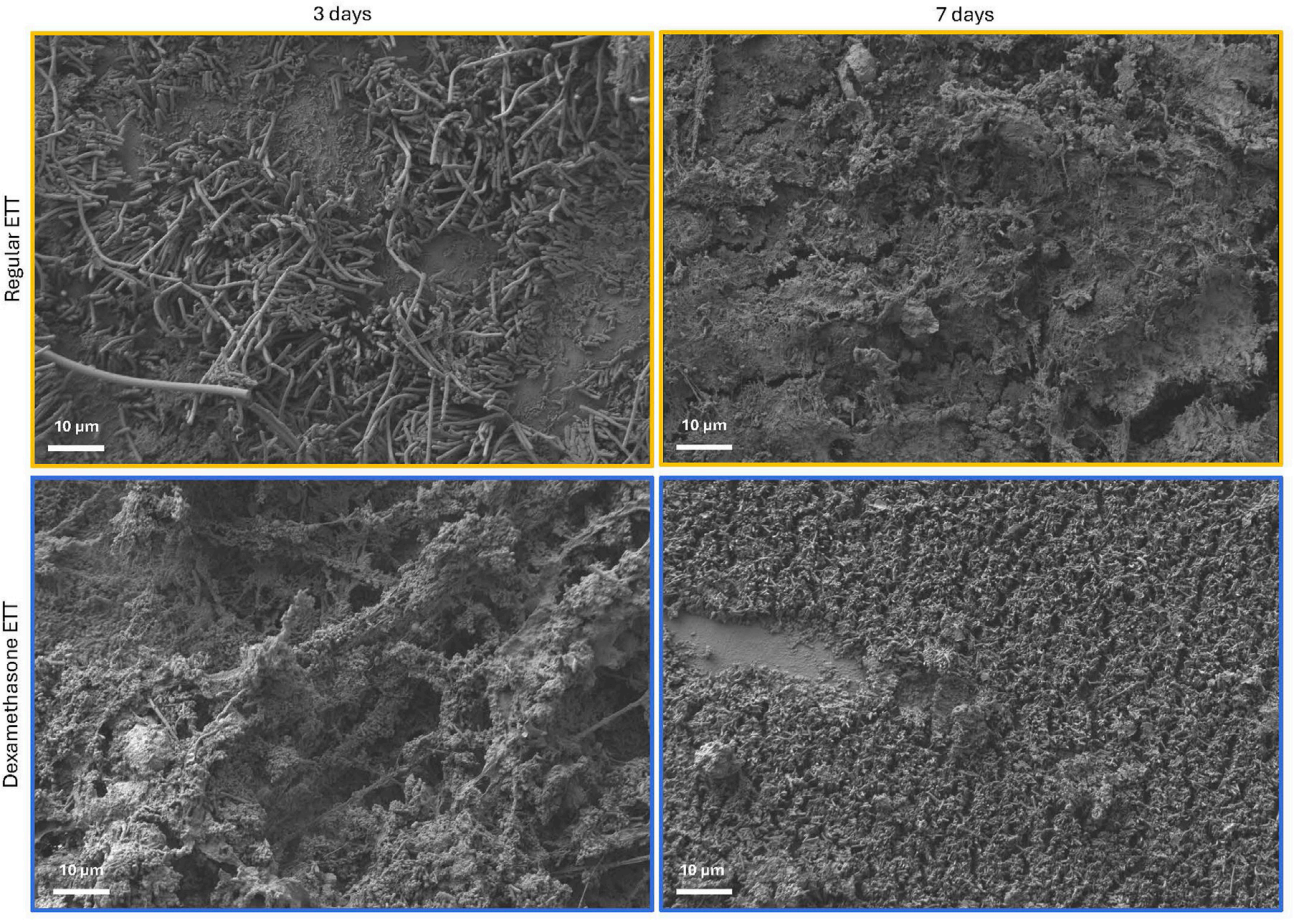
SEM micrographs of the surface of regular and dexamethasone-coated ETTs at 3 and 7 days, demonstrating biofilm formation and abundance of bacterial adhesion.

Analysis of 3D models based on µCT data revealed a trend of higher biofilm inclusions in dexamethasone coated ETTs compared to regular ETTs, though the difference did not reach statistical significance (Figure 3). Both ETT types demonstrated a trend of increase in biofilm from 3 days to 7 days. For regular ETTs, the biofilm volume increased from 39.5 ± 10.8 mm^3^ at 3 days to 91.3 ± 20.4 mm^3^ at 7 days. Similarly, dexamethasone coated ETTs exhibited an increase from 124.4 ± 25.5 mm^3^ at 3 days to 211.5 ± 90.5 mm^3^ at 7 days. The coated ETTs had a greater biofilm volume compared to regular ETTs, although differences were not statistically significant (p = 0.054). Gram stained ETT sections (Figures 3C, D) demonstrated dense clusters of bacteria, particularly surrounding the tube’s circumference. Additionally, Gram staining of the coated ETTs revealed bacteria embedded throughout the coating. SEM further confirmed biofilm formation and an abundant bacterial presence on the surface of both ETT types (Figure 4).

### Airway microbiome in the burn injured airway

Sequencing of the V3-V4 region of the 16S rRNA gene yielded an average of 75,497 reads per sample. Post-processing with the DADA2 pipeline resulted in 929,494 reads and identified 7,704 unique sequence variants. In total, 24 phyla, 39 classes, 86 orders, 143 families, and 345 genera were recognized.

**FIGURE 5:**
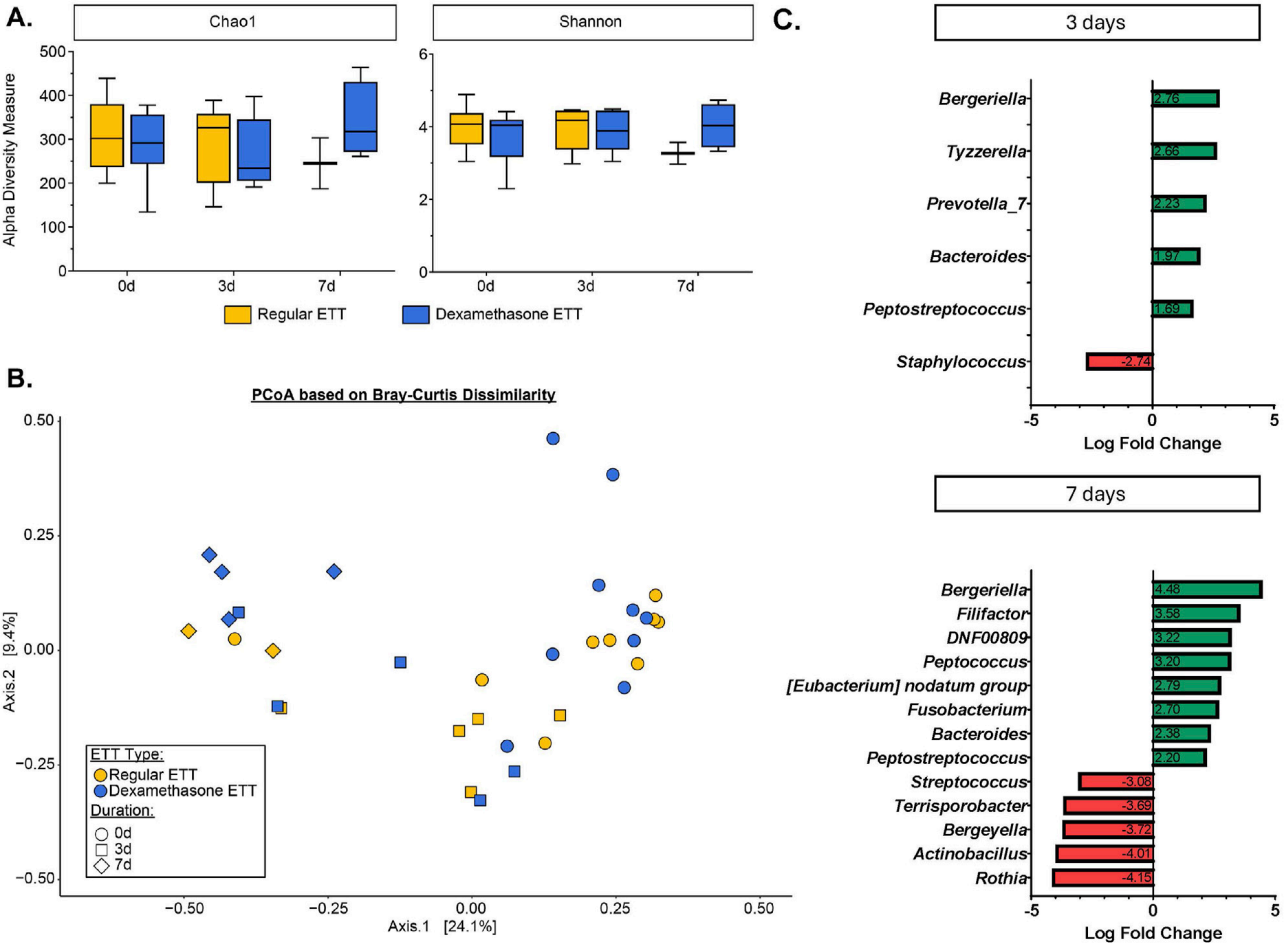
Microbial Changes in the Burn-Injured Airway **(A)** Alpha diversity measure based on Chao1 and Shannon indices. **(B)** Beta diversity analysis visualized with principal coordinate analysis (PCoA) based on Bray-Curtis dissimilarity index. **(C)** Significantly differentially abundant bacteria identified from ANCOMBC2 analysis.

Alpha diversity metrics, which measure within-sample species richness and evenness, were evaluated using the Chao1 and Shannon indices (Figure 5A). No significant differences in alpha diversity were observed between ETT types and duration of placement. The top 5 most abundant phyla in across samples were Firmicutes (39.2%), Proteobacteria (25.4%), Bacteroidota (18.8%), Fusobacteriota (10.5%), and Actinobacteriota (5.13%). At the genus level, the predominant genera were *Streptococcus* (14.2%), *Actinobacillus* (12.7%), *Porphyromonas* (11.8%), *Peptostreptococcus* (10.3%), and *Fusobacterium* (9.6%).

The PCoA based on Bray-Curtis dissimilarity accounted for 33.5% of the total variance between samples. Visualization of the plot (Figure 5B) illustrates differences in microbial composition across time points and between ETT types. These differences were statistically confirmed by the PERMANOVA test, which indicated significant variations in the microbial composition related to ETT type (R^2^ = 0.04, p < 0.05) and duration of placement (R^2^ = 0.22, p < 0.05).

Differential abundance analysis revealed no statistically significant differences between ETT types. However, significant changes were observed in several genera over time, as shown in Figure 5C. At 3 days, there were positive log fold changes in *Tyzzerella* (adj. p = 0.042) and *Prevotella_7* (adj. p = 0.042), and negative log fold changes in *Staphylococcus* (adj. p = 0.033). Additionally, positive log fold changes were observed in *Filifactor* (adj. p = 0.0007), *Peptococcus* (adj. p = 0.001), and *Fusobacterium* (adj. p = 0.008), while negative log fold changes were seen in *Streptococcus* (adj. p = 0.002), *Terrisporobacter* (adj. p = 0.011), *Actinobacillus* (adj. p = 0.002), and *Rothia* (adj. p = 0.002), among others at 7 days. Notably, *Bergeriella* (adj. p = 0.011 and adj. p < 0.0001, 3 and 7 days, respectively), *Bacteroides* (adj. p = 0.009 and adj. p = 0.009, 3 and 7 days, respectively), and *Peptostreptococcus* (adj. p = 0.049 and adj. p = 0.019, 3 and 7 days, respectively) exhibited positive log fold changes at both 3 and 7 days, with these changes increasing from early to late time points. Other significant taxa with detailed p-values are provided in Supplementary Table S1.

### Cytokine responses to regular and dexamethasone ETTs in the burn-injured airway

**FIGURE 6:**
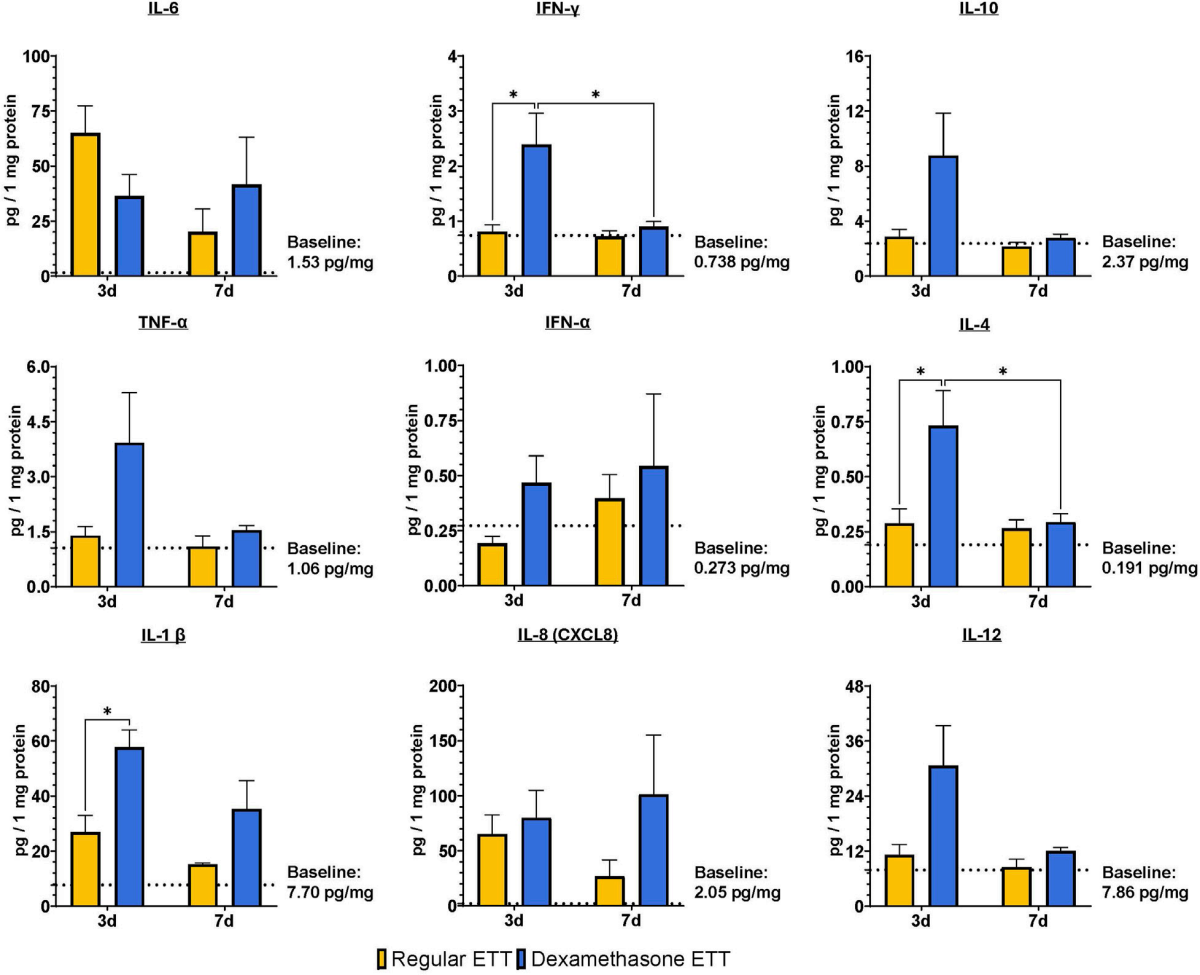
Laryngeal tissue levels of IL-6, IFN-γ, TNF-α, IFN-α, IL-1 β, CXCL8, IL-10, IL-4, and IL-12. Statistically significant differences are indicated by *<0.05, **<0.01, ***<0.001, and ****<0.0001. Baseline levels of the markers in uninjured and untreated animals are indicated by the dotted lines.

Figure 6 illustrates the evaluation of inflammatory cytokines and chemokines. Tracheal levels of IL-10, TNF-α, IFN-α, and IL-8 did not show any significant differences between ETT types or durations of placement. However, IFN-γ, IL-4, and IL-1β levels were significantly higher in the dexamethasone ETT groups compared to the regular ETT groups at 3 days (p = 0.015, p = 0.025, and p = 0.018, respectively). IFN-γ levels in the dexamethasone ETT groups significantly decreased from 2.39 ± 0.507 pg/mg protein at 3 days to 0.898 ± 0.081 pg/mg protein at 7 days (p = 0.032). Similarly, IL-4 levels significantly decreased from 0.731 ± 0.144 pg/mg protein at 3 days to 0.293 ± 0.034 pg/mg protein at 7 days (p = 0.039).

When comparing cytokine levels to control tracheal tissue without burn injury or ETT placement, IL-1β levels were significantly greater in the dexamethasone ETT groups at 3 days (57.8 ± 5.60 pg/mg protein) and 7 days (35.3 ± 8.90 pg/mg protein) in comparison to the control group (7.70 ± 3.42 pg/mg protein, p < 0.0001 and p = 0.028, respectively). Similarly, IL-12 levels were significantly higher in the dexamethasone ETT groups at 3 days (30.6 ± 7.84 pg/mg protein) compared to the control group (7.86 ± 3.40 pg/mg protein, p = 0.015). IL-6 levels were significantly higher in the regular ETT groups at 3 days (65.0 ± 11.0 pg/mg protein) compared to the control group (1.53 ± 0.340 pg/mg protein, p = 0.016).

### Macrophage marker expression in burn-injured vocal fold epithelium and vocalis muscle

**FIGURE 7:**
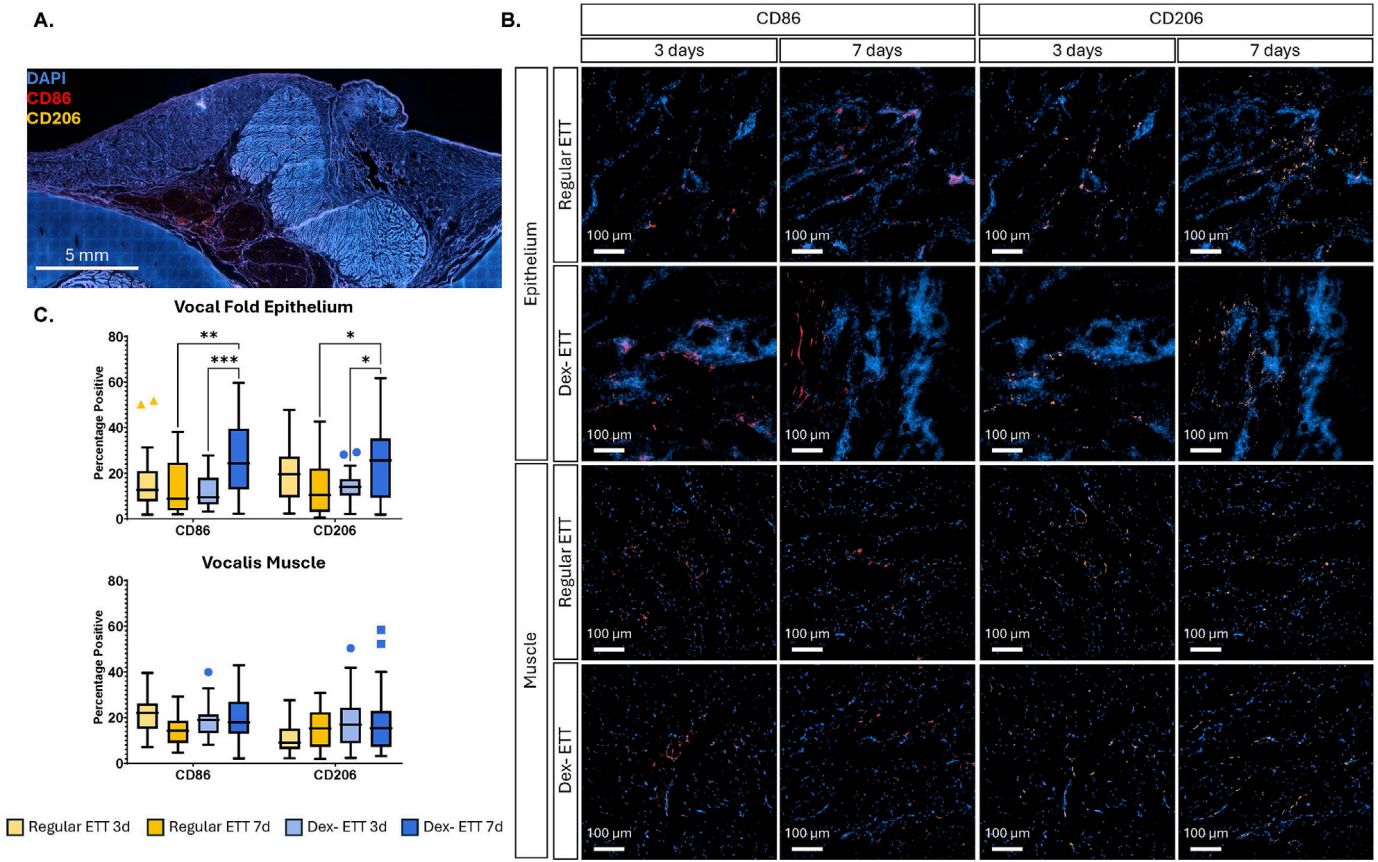
Immunostaining of CD86 and CD206 **(A)** Representative-stained vocal fold section **(B)** Regions highlighting epithelial and vocalis muscle areas, with red fluorescence indicating CD86 and orange fluorescence indicating CD206. **(C)** Quantification of surface marker expression determined from immunohistochemistry. Statistically significant differences are indicated by *<0.05, **<0.01, ***<0.001, and ****<0.0001.

We analyzed the presence of CD86 and CD206 positive cells, markers for M1 and M2 macrophages respectively (Figure 7). In the vocal fold epithelium, CD86 levels increased significantly in the dexamethasone ETT groups from 12.4% ± 1.35% at 3 days to 26.6% ± 2.99% at 7 days (p = 0.0004). Similarly, CD206 levels in the epithelium showed a significant increase in the dexamethasone groups from 14.0% ± 1.23% at 3 days to 24.7% ± 2.97% at 7 days (p = 0.0248). At 7 days, groups with dexamethasone ETT placement had significantly higher percentages of CD86 and CD206 in the epithelium compared to groups with regular ETT placement (p = 0.002 and p = 0.0213, respectively). In the vocalis muscle, there was a statistically significant interaction effect between ETT type (regular ETT at 3 days, regular ETT at 7 days, dexamethasone ETT at 3 days, and dexamethasone ETT at 7 days) and macrophage markers (CD86 and CD206) (p = 0.0053). This interaction indicates that the impact of ETT type on macrophage marker levels differs depending on the specific marker being analyzed.

### Histological analysis of epithelial thickness, percentage area of collagen, and mucins

**FIGURE 8:**
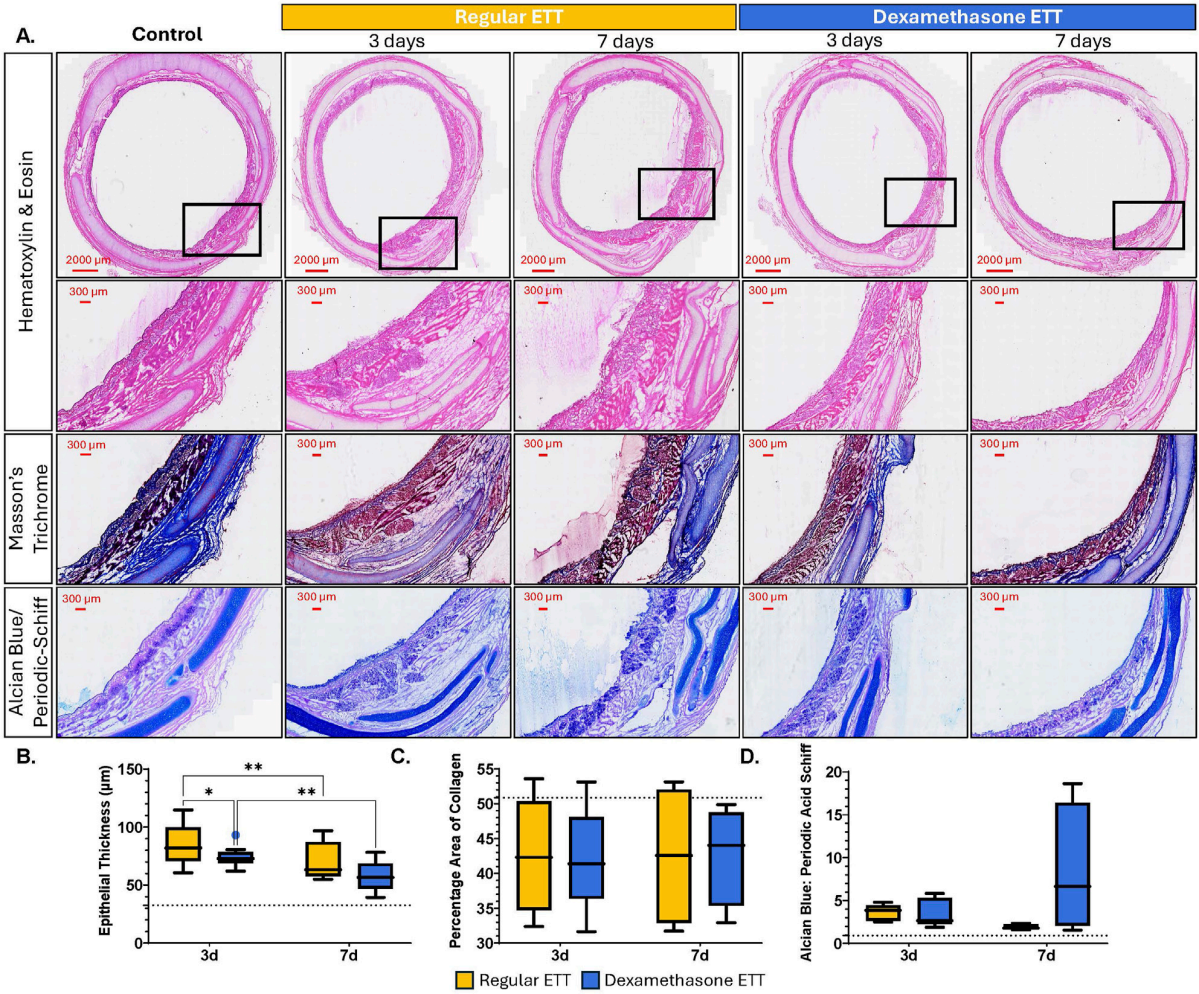
Histological Analysis of Tracheal Tissue **(A)** Cross sections of tracheal tissue stained with H&E, Masson’s Trichrome, and Alcian Blue/Periodic Acid Schiff, showing control tissue without injury or ETT placement, regular, and dexamethasone-coated ETT placement after 3 or 7 days **(B)** Quantification of epithelial thickness. **(C)** Area of collagen expressed as percentage of the total area **(D)** Alcian Blue/Periodic Acid Schiff ratio measurements. Baseline levels of the markers in uninjured and untreated animals are indicated by the dotted lines (35.5 µm epithelial thickness, 50.9% Percentage area of collagen, and 0.92 AB/PAS ratio). Statistically significant differences are indicated by *<0.05, **<0.01, ***<0.001, and ****<0.0001.

Histological evaluation of the tracheal epithelium indicated distinct morphological changes with both injury and dexamethasone treatment (Figure 8A). Epithelial thickness (Figure 8B) was significantly greater in tracheal tissue with burn injury and regular ETT placement at both 3 and 7 days (p = 0.0002 and p = 0.0167, respectively), and with dexamethasone ETT placement at 3 days (p = 0.004), compared to healthy control tissue (32.5 ± 2.44 µm). In the regular ETT groups, thickness decreased significantly from 85.5 ± 7.29 μm at 3 days to 69.7 ± 8.11 μm at 7 days (p = 0.0035). There was also a significant decrease in epithelial thickness in dexamethasone ETT groups from 73.9 ± 2.31 μm at 3 days to 57.7 ± 5.85 μm at 7 days (p = 0.0019). Additionally, epithelial thickness was significantly greater with regular ETT placement compared to dexamethasone ETT placement in the burn injured airway at 3 days (p = 0.027).

The percentage area of collagen (Figure 8C) remained consistent in the regular ETT groups, measuring 42.5% ± 3.33% at 3 days and 42.5% ± 4.37% at 7 days. Similarly, in the dexamethasone ETT groups, collagen area was 42.1% ± 3.06% at 3 days and 42.7% ± 3.11% at 7 days. These values were lower than the percentage area of collagen detected in the control tracheal tissue without injury or ETT placement (50.9% ± 5.61%), however, the differences were not statistically significant.

Alcian Blue: Periodic Acid Schiff (AB/PAS) percentage ratios (Figure 8D) were also measured to distinguish changes in acidic and neutral mucins in the tracheal tissue above the hyaline cartilage. In the regular ETT groups, AB/PAS ratio decreased from 3.62 ± 0.38 at 3 days to 1.88 ± 0.14 at 7 days. In contrast, in the dexamethasone ETT groups, the AB/PAS ratio increased from 3.56 ± 0.67 at 3 days to 8.37 ± 3.32 at 7 days. These values were higher than control trachea without burn injury or ETT placement (0.92 ± 0.32), although differences were not statistically significant.

## Discussion

Inhalation injury, resulting from inhaling extreme heat or chemicals, is a significant factor contributing to increased mortality in burn victims (Monteiro et al., 2017; Smith et al., 1994; Chen et al., 2014). Investigating the outcomes and interactions between inflammation and the microbiome in this context is essential for gaining a comprehensive understanding of the injury and providing insight into the mechanisms of laryngotracheal damage and healing. In the present study, we studied the effects of thermal injury and ETT placement on airway inflammation and microbial composition. Our approach involved the use of a drug-eluting endotracheal tube for localized delivery of dexamethasone, aiming to mitigate inflammation and prevent airway scarring.

Analysis of CT scans, particularly the surface area-to-volume (SA/V) ratios, can be a valuable metric in pathological studies for quantifying structural changes in the airway resulting from injury and noting onset or development of stenosis. Research has investigated SA/V in chronic obstructive pulmonary disease (COPD) and identified it as a potential imaging biomarker for airway remodeling, offering insight into both lung function decline and survival outcomes with disease (Bodduluri et al., 2021). The surface area-to-volume ratio significantly decreased following burn injury, as confirmed by 3D models generated from the large animal CT scans. These renderings visibly demonstrate airway narrowing, particularly where the airway conforms closely around the ETT segments placed. This reduction in the surface area-to-volume ratio reflects structural changes in the airway post-injury, likely due to swelling or other injury-related alterations affecting airway dimensions and validates the insult of burn damage induced in the current model, and establishment of airway deficit prior to ETT placement.

Studies have shown that there is an association between specific taxonomic features of airway microbiome constituents and inflammatory conditions. In cases of COPD and asthma, the phyla Firmicutes, Proteobacteria, Actinobacteria, and Bacteroidetes have been identified as predominant (Ramsheh et al., 2021; Wang et al., 2016; Leiten et al., 2020; Millares et al., 2015; Pragman et al., 2012; Tangedal et al., 2019; Marri et al., 2013; Morimoto et al., 2024). While these phyla are also dominant in a healthy human airway (Santacroce et al., 2020; Elgamal et al., 2021; Valverde-Molina and García-Marcos, 2023; Haldar et al., 2020), changes in their relative abundance, especially at the genera level, differ significantly between various respiratory conditions. Notably, *Prevotella*, *Streptococcus, Haemophilus*, and *Moraxella* are consistently prevalent when comparing differences between COPD patients and healthy controls (Ramsheh et al., 2021; Wang et al., 2016; Wang et al., 2019). These shifts in microbial composition are often driven by host inflammatory milieu and *vice versa*, making anti-inflammatory interventions particularly relevant in managing such conditions. Dexamethasone, primarily recognized for its anti-inflammatory and immunosuppressive properties, is typically used to maintain airway patency while patients are also given prophylactic antibiotics to control infection and target bacterial pathogens. Some studies have indicated that dexamethasone may reduce the efficacy of certain antibiotics, such as those targeting *Staphylococcus aureus* and *Pseudomonas aeruginosa* (Rodrigues et al., 2017). However, the combined use of corticosteroids and antimicrobial drugs has also proven to improve the effectiveness of treatment in other cases (Neher et al., 2008; Chellappan et al., 2022). While the impact of dexamethasone on changes in the microbiome and the impact of the evolving microbiome on local inflammation is anticipated, there is limited literature to identify causal relationships or specific therapeutic regimen to simultaneously target both the inflammation and potential microbial pathogenesis.

While many studies have investigated the presence of unique taxa in inflammatory airway diseases, there is a notable gap in characterizing the microbiome of the burn injured airway. An investigation of the airway microbiota after inhalation injury in burn patients revealed a significant enrichment of *Prevotella melaninogencia* and *Staphylococcus* within 3 days of injury (Walsh et al., 2017). While our study could not detect bacterial changes at the species level, we observed significant positive log fold changes for *Prevotella_7* and negative log fold changes for *Staphylococcus* at 3 days post-burn injury and ETT placement. While these changes were no longer significant after 7 days, it suggests early transient shifts in the airway microbiota with peak acute inflammation, with specific bacterial populations fluctuating in response to the initial injury and intervention.

We also observed increased log fold changes in *Bergeriella, Bacteroides*, and *Peptostreptococcus* at 3 days, with even more pronounced changes at 7 days in the burn injured airway. Some bacteria typically associated with the oral and gut microbiota can be found in other body sites, such as the respiratory tract, in cases of dysbiosis, when mucosal barriers are compromised, or accidental inhalation of oropharyngeal or gastric contents known clinically as aspiration (Marik, 2001; Imai et al., 2021; Özçam and Lynch, 2024). *Bacteroides* and *Peptostreptococcus* are both anaerobic bacteria that have previously been implicated in the pathogenesis of aspiration pneumonia (Finegold, 1995; DiBardino and Wunderink, 2015; Lode, 1988). In particular, species of *Peptostreptococcus* have been shown to increase pro-inflammatory cytokine production, such as IL-1β, in macrophages (Tanabe et al., 2007). *Bacteroides* species, typically related to the gut microbiome, have been linked to immunomodulatory polysaccharides that induce anti-inflammatory cytokines such as IL-10 which help control innate inflammatory responses under certain conditions (Ramakrishna et al., 2019; Chang et al., 2017; Mazmanian et al., 2008). While *Bergeriella* is less commonly reported, its presence in this study could indicate dysbiosis or an opportunistic infection, given its increase alongside *Bacteroides* and *Peptostreptococcus*. These microbes may favor the altered conditions present in the burn injured airway environment, potentially thriving with the presence of an ETT surface to adhere to.

Several studies have investigated the relationship between bacterial composition and the expression of inflammatory markers, revealing significant interactions between specific microbiota groups and human biomarkers of inflammation. For example, an abundance of *Moraxella* has been associated with the expression of IL-17 and TNF inflammatory pathway in patients with COPD treated with inhaled corticosteroids, while IL-8 expression has been negatively correlated with bacteria in the genera *Haemophilus*, *Moraxella*, and *Streptococcus* (Ramsheh et al., 2021; Wang et al., 2016). Although no significant differences in abundance were found for *Haemophilus* and *Moraxella*, we noted a negative log fold change in *Streptococcus* at 7 days. Additionally, IL-8 expression at 7 days was elevated in the dexamethasone-coated ETT groups compared to regular ETTs. These findings suggest similar trends to those observed in COPD studies involving corticosteroid treatment, however, further studies are needed to determine statistical causality and to explore trends correlating other bacterial strains across cytokine/chemokine secretion profiles.

In the context of burn injury, increased levels of IL-6 and IL-10 and decreased levels of IL-7 in serum samples of pediatric patients with inhalation injury have been associated with fatal outcomes (Gauglitz et al., 2008). Investigation in older populations have demonstrated that the severity of inhalation injury induces systemically measurable effects on the concentrations of IL-1RA, IL-4, IL-6, IL-7, and IL-8 in plasma. Notably, higher levels of IL-1RA and IL-6, and lower levels of IL-4 and IL-7 were observed in deceased patients compared to survivors (Davis et al., 2013). In the present study, we also observed significant shifts in the airway microbiota and corresponding changes in cytokine levels following burn injury and ETT placement. Tissues treated with dexamethasone ETTs exhibited elevated concentrations of cytokines compared to those with regular ETT placement and healthy controls. While inflammatory cytokines IL-6, IFN-α, and IL-8 increased from 3 to 7 days in the dexamethasone ETT groups, the levels of other cytokines decreased over the same period. This outcome may be contributed to an initial spike in cytokine levels at peak inflammation (3 days post-injury) due to the burn injury and the fiber-coated surface of the dexamethasone ETT, which potentially increases the inflammatory response and causes abrasion. Over time, as the dexamethasone exerts its anti-inflammatory effects, this initial inflammatory response subsides.

The immune cells in the upper airway following injury have been broadly identified including macrophages as one of the cell populations residing in the lamina propria (Catten et al., 1998; Boseley and Hartnick, 2006; Kaba et al., 2019). Polarized M1 macrophages are typically induced by pro-inflammatory cytokines such as IFN-γ and IL-6, however, they can also be stimulated by bacterial lipopolysaccharides (Shapouri-Moghaddam et al., 2018; Lendeckel et al., 2022; Chen et al., 2023; Pérez and Rius-Pérez, 2022). These macrophages produce higher levels of pro-inflammatory cytokines, including IL-6, IL-1β, TNF-α, and IL-12. In contrast, M2 macrophages are generally associated with anti-inflammatory responses producing cytokines like IL-4 and IL-10, and expressing TGF-β, which plays a key role in driving fibrosis in inflammatory conditions (Cheng et al., 2021; Ojiaku et al., 2017). However, the distinction between pro-inflammatory M1 macrophages and anti-inflammatory M2 macrophages can be an oversimplification, given the plasticity of macrophages in response to microenvironmental stimuli such as biofilm and ETT placement (Strizova et al., 2023). In our study, the significant increase in both CD86 and CD206 markers in the vocal fold epithelium in dexamethasone groups over time and compared to regular ETT groups at 7 days likely reflects the increased biofilm formation observed on the coated ETTs. This conclusion is supported by the inclusions observed in 3D composites constructed from the µCT and SEM which showed extensive bacterial adhesion on the surface, indicating interplay between biofilm formation and macrophage response.

We further investigated the structural changes in the trachea to better understand the tissue remodeling and healing process post burn-injury and ETT placement. Epithelial thickness measurements indicated an increase in the trachea epithelium after burn injury and ETT placement compared to control tissue, likely corresponding to epithelial hyperplasia. The significant reduction in tracheal epithelial thickness in both ETT types from 3 to 7 days aligns with the expected healing process, as the tissue gradually returns to its native state. Our group has previously explored epithelial ulceration, inflammation, and fibrosis in laryngeal tissue following inhalational burn injury and found minimal changes in degree of fibrosis and ulceration from 3 to 7 days, with both regular and dexamethasone ETTs, though inflammation scores were higher in the dexamethasone-coated ETTs compared to regular ETTs (Malka et al., 2023). Regarding the remodeling phase of collagen, there were no significant differences observed in the percentage area of collagen between the different ETT types. ETT groups showed a reduced collagen percentage compared to the healthy control. While an increase in collagen deposition is expected as part of the tissue’s normal wound healing process, the time frame of the study may not have been sufficient for collagen accumulation. It is also possible that the increased epithelial thickening serves as a compensatory mechanism to protect against a weakened collagen matrix that is affected by injury and ETT placement.

The airway surfaces are lined with a protective layer of mucus that is continuously renewed to aid in the clearance of foreign pathogens and maintain respiratory health (Song et al., 2020; Pangeni et al., 2023). However, in conditions such as inhalation injury or with the placement of an ETT, the production of these mucins can be disrupted leading to an imbalance in mucus composition that impairs the mucociliary clearance and increases the risk of infection (Powell et al., 2018; Cox et al., 2008; Cox et al., 2003; Koeppen et al., 2013). Our study observed the AB/PAS ratio to have increased levels of acidic mucins in tracheal tissue with ETT placement compared to control tissue. The subsequent reduction in the AB/PAS ratio over time suggests a shift towards a more stabilized mucosal environment as the initial inflammatory response subsides. Interestingly, the increased ratio observed in the dexamethasone ETT groups implies the use of the anti-inflammatory fiber coating is resulting in the production of more mucins, likely as the airway attempts to protect itself and facilitate the removal of the irritant. There has also been evidence that the inclusion of lubricating coatings on endotracheal tubes can lead to the further retention of epithelial mucosa and its secretory function (Miar et al., 2024).

There are several limitations to our study that merit consideration. The duration of the study was insufficient to fully capture the long-term effects of burn injury and ETT placement on airway healing and microbial composition changes. Although our injury model is validated across studies, variations in burn severity and the small sample size may have influenced the study outcomes and limited our ability to identify statistically significant correlations between microbial shifts and cytokine levels. Additionally, findings from animal studies may not directly translate to human patients given the profound differences in the native microbiome. Further research is necessary to understand the implications of these findings in clinical settings.

## Conclusion

This study reveals that burn injury and ETT placement significantly impact airway inflammation, structure, and microbiome. While both regular and dexamethasone-eluting ETTs affect the laryngotracheal environment, dexamethasone ETTs increased biofilm inclusions, certain inflammatory cytokines, and mucin production, possibly due to initial irritation from the ETT electrospun fiber coating. Additionally, there were notable shifts in the microbial community with burn injury, ETT type, and duration of placement, with significant differences in specific taxa observed at various time points. Future studies will focus on optimizing our drug-delivery technologies and strategies for managing burn-injured patients.

## Data Availability

All data generated during and/or analyzed during the current study are available in Supplementary Information file #2. Sequencing data is available in a publicly accessible repository. This data can be found here: https://www.ncbi.nlm.nih.gov/, BioProject ID PRJNA1226158. Further inquiries can be directed to the corresponding author.
